# The diagnostic and prognostic value of H2AFY in hepatocellular carcinoma

**DOI:** 10.1186/s12885-021-08161-4

**Published:** 2021-04-15

**Authors:** Xuyang Ma, Ying Ding, Li Zeng

**Affiliations:** 1 Department of Gastroenterology, Luzhou People’s Hospital, Section 2 of JiuGu Avenue, Jiangyang District, Luzhou, 646000 Sichuan People’s Republic of China; 2 Department of Technology Education Training, Luzhou People’s Hospital, Section 2 of JiuGu Avenue, Jiangyang District, Luzhou, 646000 Sichuan People’s Republic of China

**Keywords:** H2AFY, MacroH2A1, Hepatocellular carcinoma, Bioinformatics

## Abstract

**Background:**

The potential correlation between H2AFY (also known as MacroH2A1) and the clinical characteristics of hepatocellular carcinoma (HCC) patients was analysed through gene expression profiles and clinical data in The Cancer Genome Atlas (TCGA) database, and the diagnostic and prognostic value of H2AFY in HCC was discussed.

**Methods:**

The gene expression data of HCC and the corresponding clinical characteristics of HCC patients were downloaded from the TCGA database. The differences in H2AFY in normal liver tissues and HCC were analysed. The relationship between H2AFY and clinical characteristics was analysed by Wilcoxon signed-rank test, logistic regression and Kruskal-Wallis test. The Kaplan-Meier method and the Cox regression method were used to analyse the relationship between overall survival and clinical characteristics of the patients. An ROC curve was used to predict the diagnostic value of H2AFY in HCC. Gene set enrichment analysis (GSEA) was used to analyse the pathway enrichment of H2AFY.

**Result:**

Compared with normal liver tissues, H2AFY was significantly highly expressed in HCC. H2AFY was positively correlated with the age, clinical stage, G stage (grade) and T stage (tumor stage) of liver cancer patients. Higher H2AFY expression predicted a poor prognosis in HCC patients. Cox regression analysis suggested that H2AFY was an independent risk factor for the prognosis of HCC patients. The ROC curve suggested that H2AFY had certain diagnostic value in HCC. GSEA suggested that H2AFY was correlated with lipid metabolism and a variety of tumour pathways.

**Conclusion:**

Our study showed that H2AFY was significantly overexpressed in HCC. H2AFY may be a potential diagnostic and prognostic marker for HCC, and high expression of H2AFY predicts a poor prognosis in patients with HCC.

## Background

Hepatocellular carcinoma (HCC) is the most common malignant tumour, usually occurs during the terminal stage of cirrhosis and is the third leading cause of cancer-related death [1, 2]. Presently, the most commonly used monitoring method for HCC is ultrasonography, which has high specificity and sensitivity. However, in actual clinical practice, the accuracy of ultrasonography is limited by the level of operating physicians [3, 4]. AFP (α-fetoprotein) is the most commonly used serum tumour marker for the diagnosis of HCC, but a retrospective study shows that the sensitivity of AFP in clinical practice is only approximately 60%, while its specificity is 80% [5, 6]. The combination of ultrasound and AFP is not more advantageous, as it increases false positives and costs [3, 7]. Surgery for HCC is the most common treatment, but most patients experience poor therapeutic effects and short postoperative survival [2, 8]. The discovery of new targets and molecules at the gene level may be an effective method to improve outcomes.

H2AFY (also known as MacroH2A1) is a member of the core histone H2A family, the other members of which include H2A. X, H2A. Z, and H2A. Bb [9]. The H2A family has been shown to be abnormally expressed in a variety of tumours [10]. H2AFY is an atypical histone that has a large macro domain and can interact with a variety of molecules [11]. According to different splicing methods, H2AFY can be alternatively spliced into two isoforms, MacroH2A1.1 and MacroH2A1.2 [9]. Although the overall expression level of H2AFY did not change, the protein-coding gene QKI promoted the expression of MacroH2A1.1, while the RNA helicases DDX5/DDX17 promoted the expression of MacroH2A1.2 [12, 13]. MacroH2A1.1 and MacroH2A1.2 differ in their functional macro domains [14], and they are significantly different from each other [15, 16].

In normal cells, H2AFY, as part of chromatin, can affect gene expression and silencing by regulating transcriptional activation [17]. H2AFY has been shown to be involved in the development of a variety of tumours, as it plays a corresponding role in breast, lung and colon cancers [9]. Most studies suggest that H2AFY is involved in the process of tumour suppression [18], but sometimes it exerts the opposite effect [19, 20].

In some studies, H2AFY exhibited anticancer properties. H2AFY has a negative regulatory relationship with the differentiation of pluripotent stem cells [21], and is thus considered a marker of highly differentiated hepatocyte tumour cells [22]. The loss of H2AFY mediates the phosphorylation level of the NF- κBp65 (Ser536) pathway, which induces hepatoma cells to exhibit stemness [22]. In HCC, as tumour cells exhibit stem cell-like properties, the loss of H2AFY can change the glucose metabolism and lipid metabolism in HCC cells, so that tumour cells can obtain energy and intermediate metabolites, which is beneficial for those cells to adapt to their changing microenvironment [23]. Inhibition of H2AFY induces tumorigenicity and expression of the transcription factor ZEB1, which leads to a poor prognosis in patients with colon cancer [24]. However, in some studies, H2AFY has been shown to be carcinogenic. H2AFY is an immunohistochemical marker of HCC, which together with DNA hypomethylation, mediates and attenuates the senescence process of HCC cells and promotes HCC progression [19]. When DNA is demethylated, the deletion of H2AFY enhances the reactivation of the tumour suppressor genes p16, MLH1, and Timp3, thereby inhibiting cell proliferation [20].

Although previous studies have confirmed the high expression of H2AFY in HCC [19], whether H2AFY can play a role in clinical practice was unknown. We believe that it is necessary to study the correlation between H2AFY and the clinical characteristics of patients with HCC, and to further explore the application value of H2AFY in clinical practice.

## Methods

### Collection of genetic and clinical data

Gene expression profile data of HCC patients were downloaded from the TCGA database (https:// portal.gdc.cancer.gov/repository), which included 50 samples of normal liver tissues and 374 HCC tissues (Workflow Type: HTSeq-FPKM). Data on the clinical characteristics of 377 patients with HCC were also collected from the TCGA. Then, the expression of H2AFY in normal liver and liver cancer tissues was demonstrated by Boxplots and a paired differential plot.

### Gene set enrichment analysis

GSEA was used to determine whether the target gene was differed between the normal liver sample and HCC tissues [25]. GSEA software was used to perform a pathway enrichment analysis on H2AFY according to the instructions on the GSEA website (version: GSEA-v3.0; https://www.gsea-msigdb.org/gsea/index.jsp). For each analysis, 1000 gene set permutations were performed. According to the normalized enrichment score (NES), the significantly enriched gene sets were screened. Then, the enrichment pathways with a normal *p*-value < 0.05 and a false discovery rate (FDR) < 0.25 were selected. MSigDB: c2.cp.kegg.v7.0.symbols.gmt.

### Statistical analysis

R was used for statistical analysis (version: R × 64 v3.6.2). The Kruskal-Wallis test (multiple continuous independent samples), Wilcoxon rank test (two continuous independent samples) and logistic regression were used to analyse the relationship between H2AFY and the clinical characteristics of HCC. Patient data with incomplete clinical information were omitted in advance. Cox regression analysis was then used to compare the effects of H2AFY and clinical characteristics on overall survival. The ROC curve (receiver operating characteristic curve) was plotted using SPSS 19.0. The cut-off value for H2AFY expression was determined by the middle bit value.

## Result

### Clinical characteristics of patients with hepatocellular carcinoma

The clinical characteristics of 377 patients with HCC were collected from the TCGA. However, the clinical information of some patients was unavailable. We provide detailed information of these clinical features in Table 1.

**Table. 1:** The Clinical characteristics of hepatocellular carcinoma patients obtained from TCGA database

Clinical characteristics	Total (377)	%
Age	61 (16–90)	
G stage		
G1	55	14.82
G2	180	48.51
G3	124	33.42
G4	12	3.25
Clinical stage		
Stage I	175	49.58
Stage II	87	24.65
Stage III	86	24.36
Stage IV	5	1.41
Sex		
Female	122	32.36
Male	255	67.64
T stage		
T1	185	49.47
T2	95	25.40
T3	81	21.66
T4	13	3.47
N stage		
N0	257	98.46
N1	4	1.54
M stage		
M0	272	98.55
M1	4	1.45

### The relationship between H2AFY and the clinical characteristics of hepatocellular carcinoma patients

**Fig. 1:**
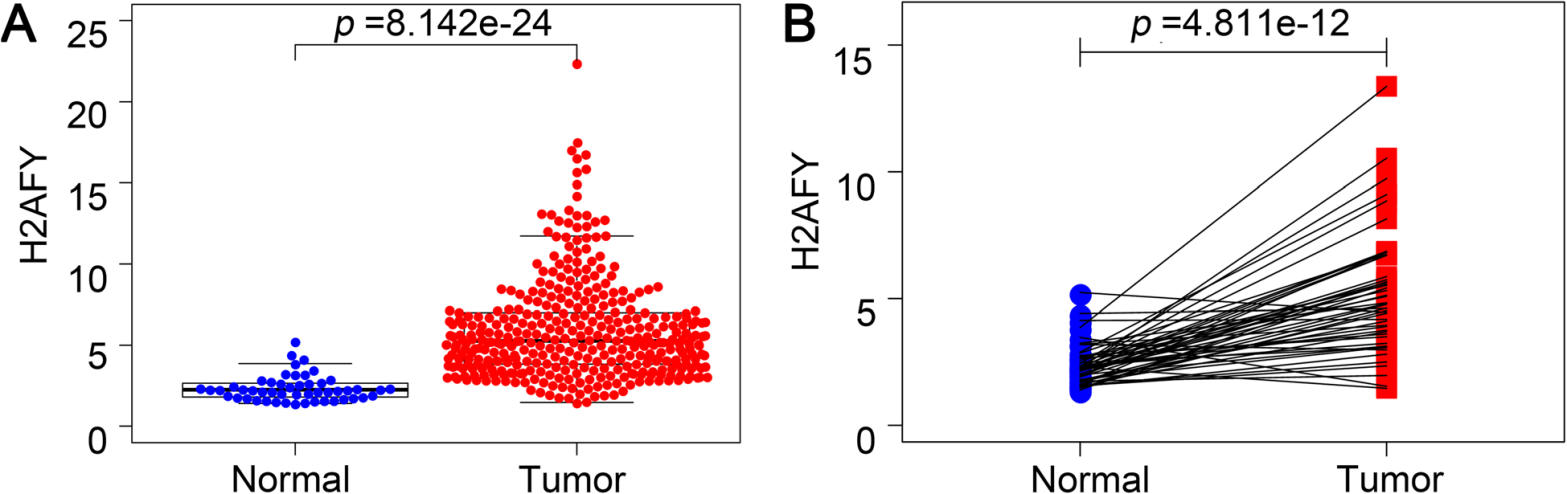
The expression level of H2AFY in HCC and normal liver tissues. H2AFY is more highly expressed in HCC compared with normal liver tissues

Compared with normal liver tissues, H2AFY is more highly expressed in HCC, as shown in boxplots and the paired differential plot (see Fig. 1a and b, *p* < 0.001).

**Fig. 2:**
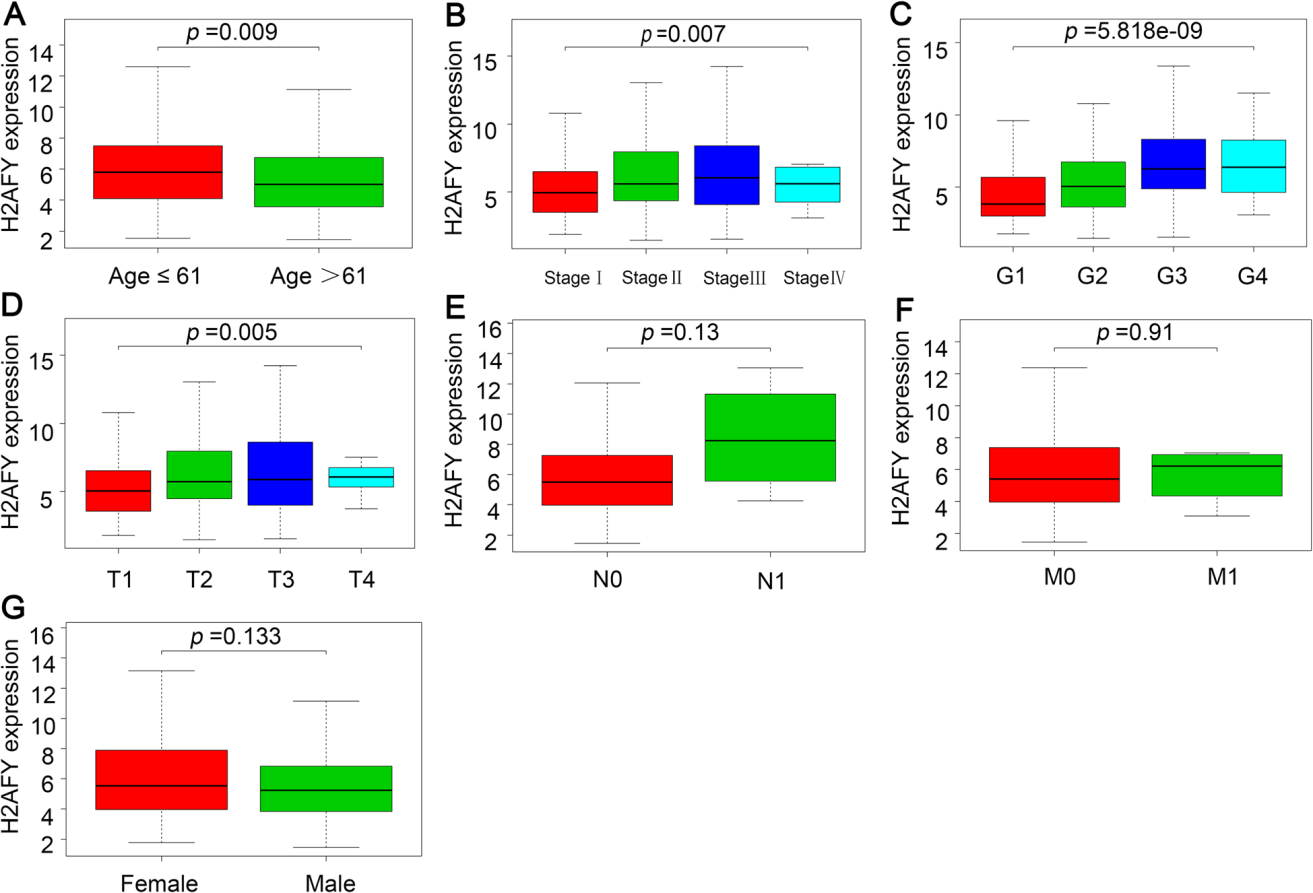
The relationship between H2AFY expression and clinical characteristics. **a**. age. **b** clinical stage. **c** grade. **d** tumor stage. **e** lymph node metastasis. **f** distant metastases. **g** sex

Figure 2a-g shows the relationship between H2AFY and the clinical characteristics of the patients. The results showed that the expression level of H2AFY in HCC was positively correlated with the patient’s age (*p* = 0.009), clinical stage (*p* = 0.007), G stage (*p* = 5.818e-09) and T stage (*p* = 0.005), while no significant correlation was observed between H2AFY and N stage, M stage or the patient’s sex. Logistic regression was used to further analyse the relationship between H2AFY and the clinical characteristics of HCC patients and suggested the same result. As clinical stage, histological grade and T stage progressed, the expression level of H2AFY in HCC gradually increased, as shown in Table 2.

**Table. 2:** Logistic regression of H2AFY expression and clinical characteristics

Clinical characteristic	Total (N)	Odds ratio of H2AFY expression	95%CI	***p***-value
Age (>61vs ≤ 61)	376	0.57	0.37–0.86	0.007
Clinical stage (Stage III vs Stage I)	256	1.78	1.05–3.03	0.030
G stage				
(G2 vs G1)	235	1.96	1.02–3.90	0.048
(G3 vs G1)	179	5.53	2.77–11.49	2.0857e-06
(G4 vs G1)	68	5.20	1.42–21.97	0.015
T stage (T4 vs T1)	198	4.30	1.26–19.66	0.030
N stage (N1 vs N0)	261	3.04	0.38–62.07	0.337
M stage (M1 vs M0)	271	3.04	0.38–61.99	0.337
Sex (Male vs Female)	377	0.83	0.54–1.28	0.417

CI: confidence interval

### Survival analysis and clinical diagnostic efficacy of H2AFY

**Fig. 3:**
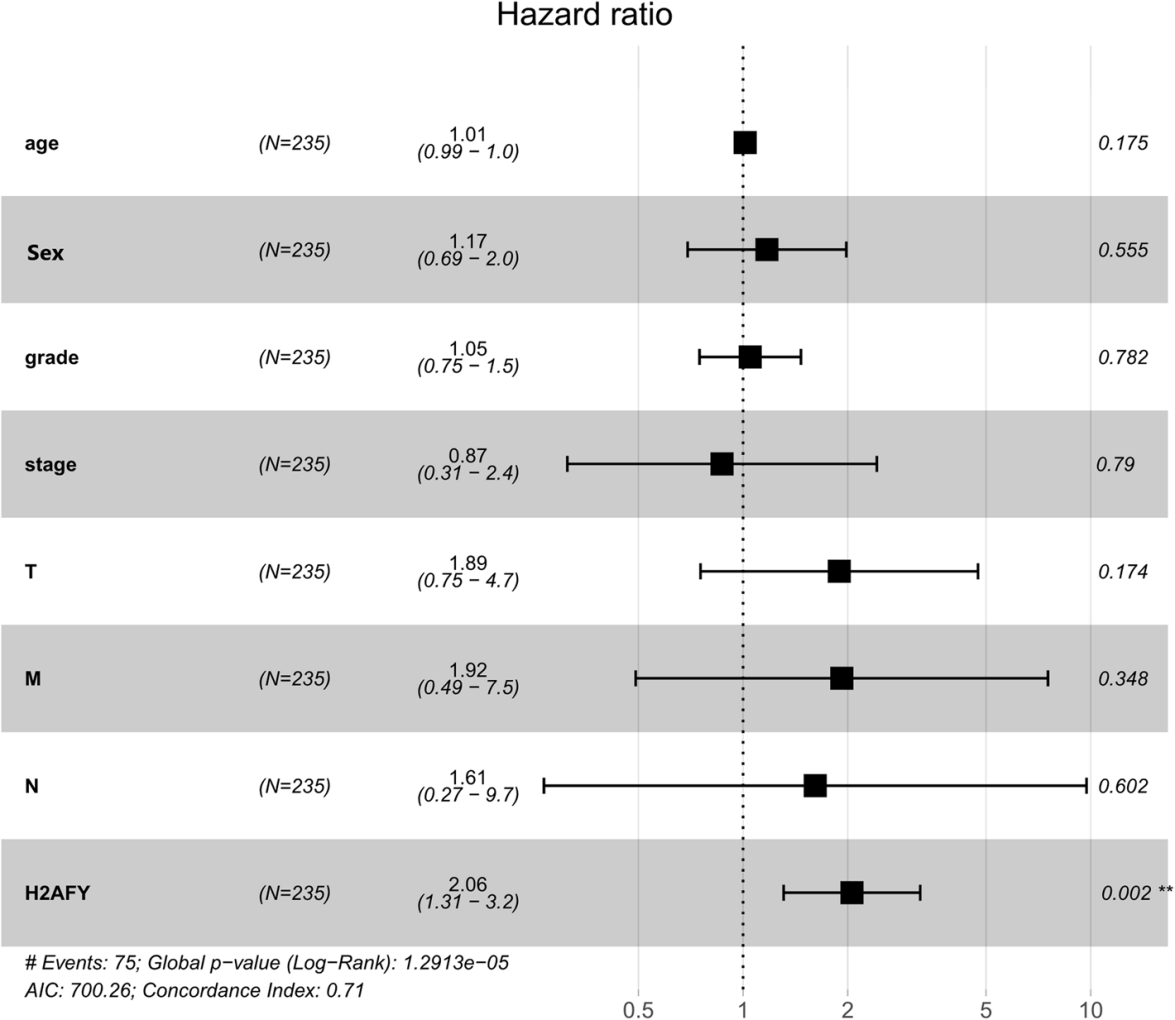
The forest plot shows the multivariate analysis of the relationship between H2AFY expression and overall survival among HCC patients

**Fig. 4:**
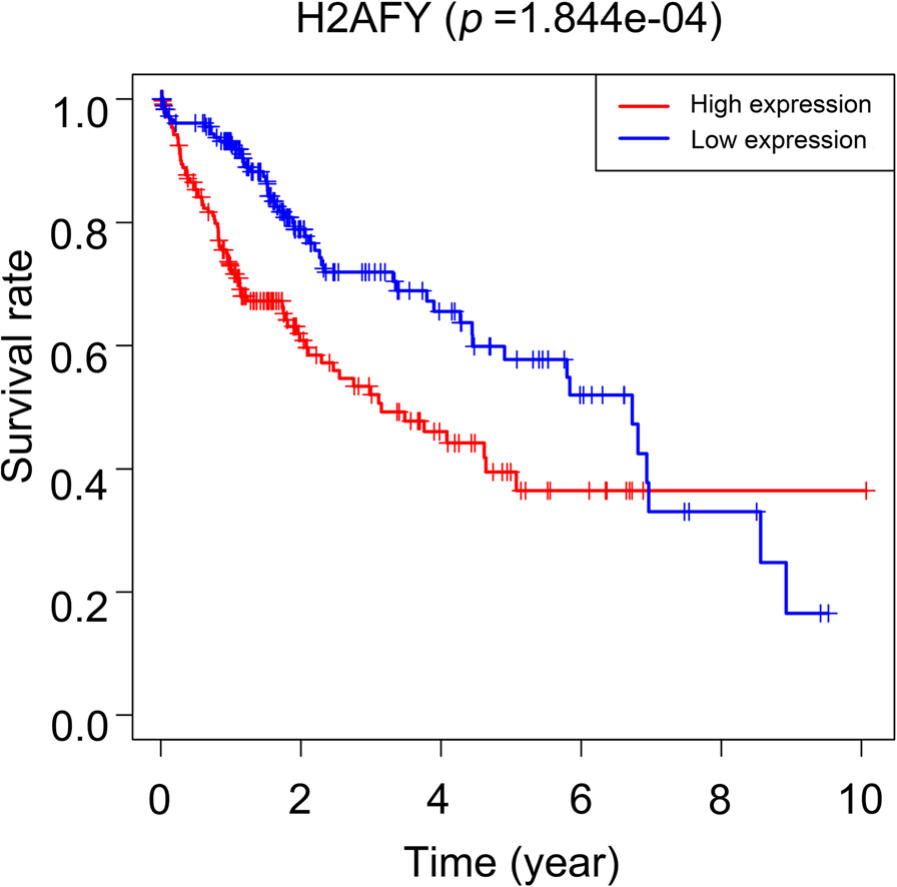
The relationship between H2AFY expression and overall survival in HCC patients

**Fig. 5:**
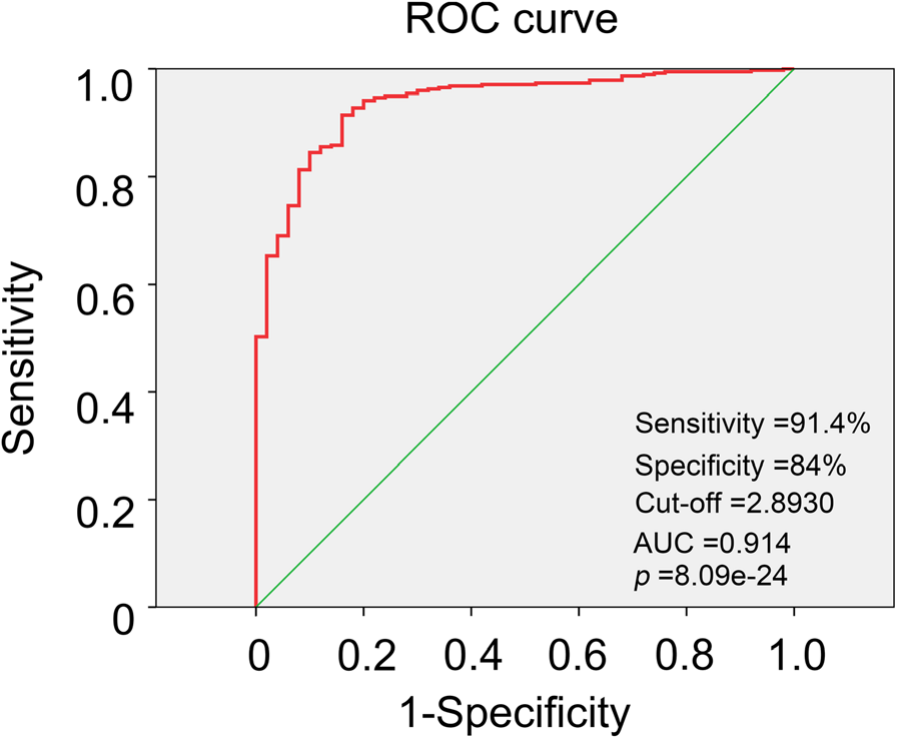
The ROC curve demonstrated the diagnostic value of H2AFY in HCC patients

Cox regression and Kaplan-Meier methods were used to analyse the potential relationship between H2AFY and the overall survival of patients. The results of the univariate Cox analysis suggested that H2AFY was a high-risk factor for HCC (HR: 2.298, CI: 1.533–3.443, *p* = 5.492e-05), as shown in Table 3. Using the forest plot to demonstrate the results of the multivariate Cox analysis, we found that H2AFY was an independent risk factor for the prognosis of patients with HCC (HR: 2.056, CI: 1.308–3.232, *p* = 0.001), as seen in Fig. 3. The results of the Kaplan-Meier analysis showed that HCC patients with higher H2AFY expression had a lower 5-year survival rate, as shown in Fig. 4. The ROC curve analysis results showed that the area under the ROC curve of H2AFY was 0.914 (CI: 0.907–0.968, *p* = 8.09e-24) and that the sensitivity and specificity of H2AFY in differentiating HCC from normal liver tissues were 91.4 and 84%, respectively (Fig. 5). These results suggest that H2AFY can be used as a diagnostic and prognostic marker for HCC.

**Table 3:** Univariate Cox analysis of the relationship between H2AFY expression and overall survival among hepatocellular carcinoma patients

Clinical characteristic	HR	%95CI	***p***-value
H2AFY	2.298	1.533–3.443	5.492e-05
Clinical stage	1.864	1.455–2.388	8.066e-07
T stage	1.804	1.455–2.270	4.725e-07
M stage	3.850	1.206–12.281	0.022
N stage	2.021	0.493–8.276	0.327
G stage	1.017	0.745–1.387	0.914
Sex	0.780	0.487–1.249	0.301
Age	1.005	0.986–1.023	0.591

HR: hazard ratio; CI: confidence interval

### GSEA enrichment analysis results of H2AFY

**Fig. 6:**
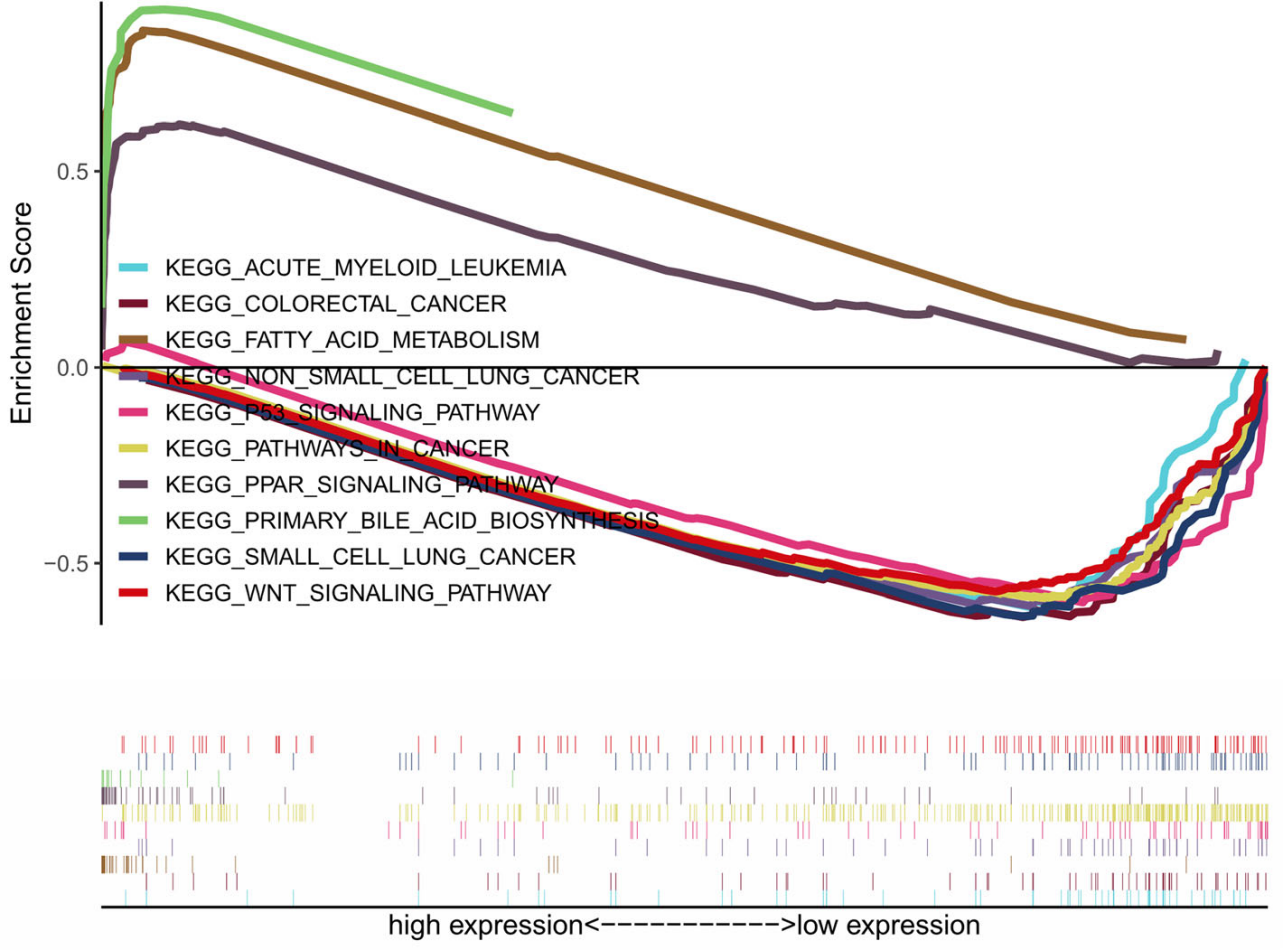
Enrichment plots from gene set enrichment analysis (GSEA)

**Fig. 7:**
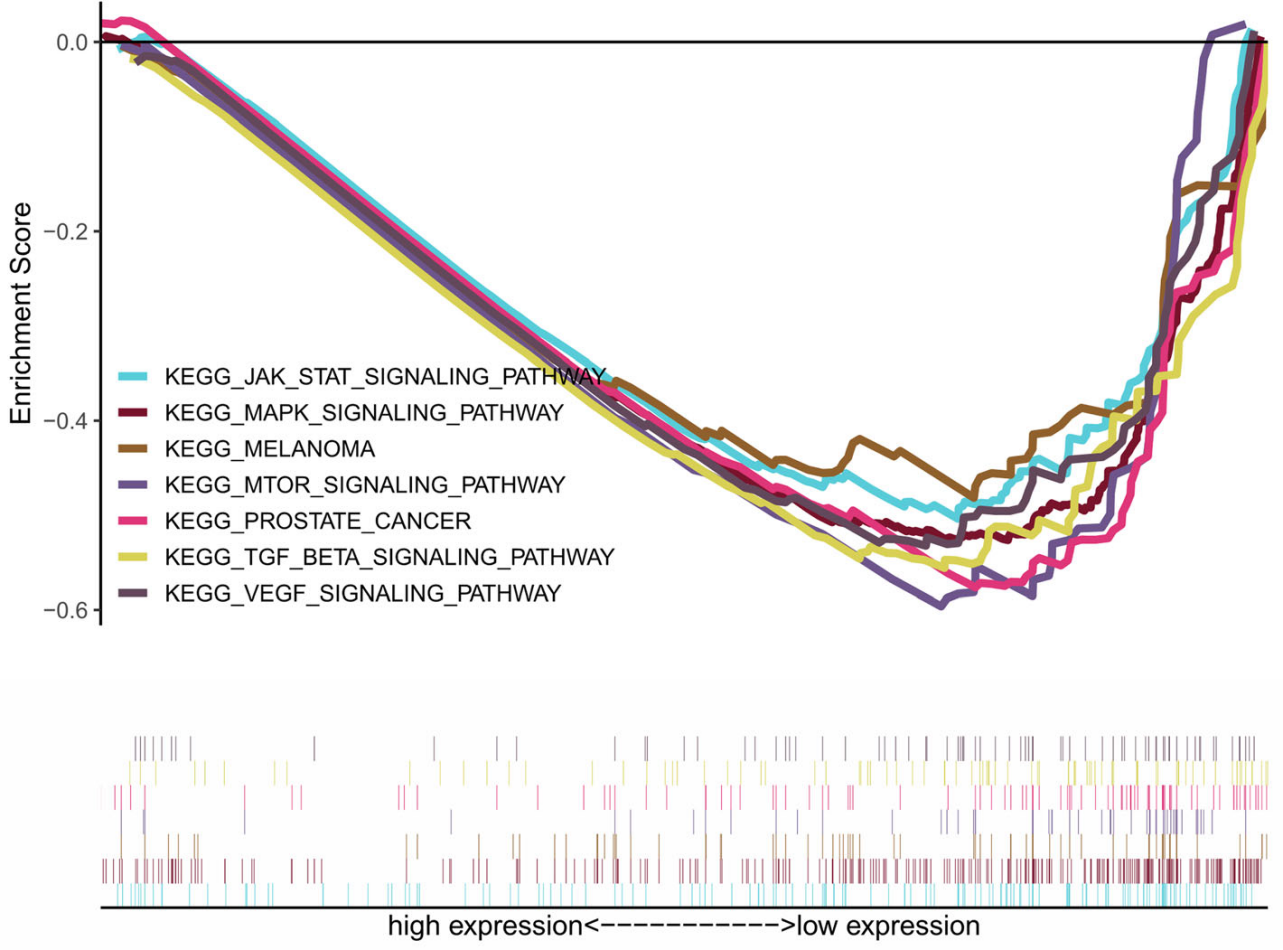
Enrichment plots from gene set enrichment analysis (GSEA)

In order to preliminarily explore the possible ways and pathways through which H2AFY functions in the development of HCC, we used GSEA to perform an enrichment analysis on H2AFY. According to the *p*-value < 0.05, FDR < 0.05 and NES, significant enrichment pathways were screened, and the results are shown in Table 4, Fig. 6, and Fig. 7. The results demonstrate that fatty acid metabolism, cell cycle, PPAR signalling pathway, pathways in cancer, p53 signalling pathway, Wnt signalling pathway, MAPK signalling pathway, TGF beta signalling pathway, melanoma, prostate cancer, acute myeloid leukaemia and others are correlated with H2AFY.

**Table 4:** GSEA gene enrichment results of H2AFY

Gene set	NES	***p***-value	FDR
FATTY_ACID_METABOLISM	2.179	0.000	0.000
PRIMARY_BILE_ACID_BIOSYNTHESIS	2.091	0.000	2.84e-04
PPAR_SIGNALING_PATHWAY	2.019	0.000	8.14e-04
PATHWAYS_IN_CANCER	−1.989	0.000	0.003
P53_SIGNALING_PATHWAY	−1.979	0.000	0.003
SMALL_CELL_LUNG_CANCER	−1.922	0.000	0.005
WNT_SIGNALING_PATHWAY	−1.898	0.000	0.007
COLORECTAL_CANCER	− 1.893	0.000	0.007
ACUTE_MYELOID_LEUKEMIA	−1.892	0.000	0.007
NON_SMALL_CELL_LUNG_CANCER	−1.890	0.000	0.007
PROSTATE_CANCER	−1.845	0.000	0.008
MAPK_SIGNALING_PATHWAY	−1.833	0.000	0.009
mTOR_SIGNALING_PATHWAY	−1.824	0.001	0.009
VEGF_SIGNALING_PATHWAY	−1.789	0.003	0.012
TGF_BETA_SIGNALING_PATHWAY	−1.722	0.005	0.018
MELANOMA	−1.581	0.006	0.049
JAK_STAT_SIGNALING_PATHWAY	−1.681	0.013	0.025

## Discussion

In recent years, more and more studies have shown that H2AFY is differentially expressed and plays corresponding biological functions in multiple tumour types, including HCC, lung cancer, prostate cancer, acute myeloid leukaemia (AML), melanoma, and colon cancer [15, 26–30]. According to previously published studies, the role of H2AFY in tumours is very complex and it is very challenging to study H2AFY in tumours. H2AFY has been reported to function in HCC stemness, in highly differentiated HCC, and steatosis-related HCC, but the clinical application value of H2AFY has not yet been reported. Therefore, this study focused on the exploration of the relationship between H2AFY and the clinical characteristics of HCC, and clarified the correlation between H2AFY and the prognosis of HCC patients.

Here, we conducted a preliminary study on the expression of H2AFY in patients with HCC using gene expression profile data and clinical feature data in the TCGA database. Compared with normal liver tissues, the expression level of H2AFY in HCC is higher, and as age, clinical stage, histological grade, T stage and HCC progress, the expression level of H2AFY in HCC shows a gradually increasing trend. The results of the survival analysis and logistic regression were also consistent with the above results, which suggests that the patients with high expression of H2AFY had lower survival and a poor prognosis. The results of the multivariate Cox analysis suggested that H2AFY was an independent risk factor for HCC, and the ROC curve suggested the potential value of H2AFY in the diagnosis of HCC. Therefore, H2AFY may play an important role in the development of HCC, and H2AFY is helpful for diagnostic and prognostic analyses of HCC patients.

Our analysis results based on the TCGA database showed that H2AFY was highly expressed in HCC, which suggests that H2AFY may promote the occurrence and development of HCC. However, most studies have suggested that H2AFY plays a role in tumour suppression [15, 21], which seems to contradict our results. However, some studies have reported that H2AFY has a carcinogenic role [13, 20, 31]. Actually, H2AFY has two different splice variants (MacroH2A1.1 and MacroH2A1.2), which are quite different in function. Different expression rates of the two isoforms of H2AFY in tumours may lead to different outcomes, which we believe may provide part of the explanation.

The expression ratio of MacroH2A1.1 and MacroH2A1.2, the two isoforms of H2AFY, may be of great significance. MacroH2A1.1 acts as a tumour suppressor by inhibiting cell proliferation, migration, and invasion, while the function of MacroH2A1.2 is largely dependent on the type of cancer [18, 32]. In differentiated and proliferating cells, MacroH2A1.1 and MacroH2A1.2 have obvious expression differences: MacroH2A1.1 is primarily found in differentiated cells, while MacroH2A1.2 is mainly found in proliferating cells [33, 34].

Most studies have confirmed that MacroH2A1.1 exerts anti-cancer effects [12, 13]. MacroH2A1.1 plays an anticancer role in prostate cancer and reduces tumour malignancy [26]. Macroh2a1.1 can inhibit Epithelial Mesenchymal Transition (EMT) [35]. The unbalanced expression of H2AFY isoforms, especially the reduction in MacroH2A1.1, will lead to the impaired differentiation of red blood cells, and will eventually lead to anaemia in myelodysplastic syndrome (MDS) patients [36]. In colon cancer patients, MacroH2A1.1 mRNA expression was decreased, while MacroH2A1.2 mRNA expression was up-regulated. MacroH2A1.1 expression was negatively correlated with disease severity and survival, while MacroH2A1.2 did not show that same characteristic [30]. However, sometimes MacroH2A1.1 also shows the opposite effect, and in one study, high expression of MacroH2A1.1 was associated with poor prognosis in triple-negative breast cancer [37]. Compared with MacroH2A1.1, MacroH2A1.2 has a more complex role in tumours. MacroH2A1.2 is overexpressed in tumour cells, and its macro domain interacts with HER-2 to promote the proliferation and carcinogenicity of cancer cells [31]. In tumour xenografts, MacroH2A1.2 increased the invasiveness, growth and migration of cancer cells [13]. In both in vivo and in vitro experiments, reducing the expression level of MacroH2A1.2 could promote the progression of melanoma by increasing the expression of the CDK8 oncogene [29].

Three known splicing factors have been described for H2AFY, including splicing factor MBNL1, the protein-coding gene QKI, and the RNA helicases Ddx5 and Ddx17 [12, 38]. The former two promote the expression of MacroH2A1.1, while the latter are beneficial to the expression of MacroH2A1.2. These splicing factors can produce two alternative transcripts. At the same time, some studies have shown that the two isoforms may have similar domains and that both isoforms are responsible for X chromosome inactivation [39].

Based on previous studies, we hypothesized that there might be a competitive association between the two isoforms of H2AFY. MacroH2A1.1 has cancer-inhibiting properties, while MacroH2A1.2 has cancer-promoting characteristics, and both of them jointly regulate the occurrence and development of tumours. Therefore, we attempted to further determine the expression levels of the two H2AFY isoforms in HCC. Unfortunately, we were unable to retrieve the respective expression data of the two isoforms of H2AFY from the TCGA database. However, our study raises the possibility that in patients with HCC, the diagnosis and prognosis of HCC may be assessed based on the total expression of H2AFY alone, rather than the expression of the H2AFY isoform.

We conducted an enrichment analysis of H2AFY by GSEA. The analysis results showed that H2AFY may be correlated with fatty acid metabolism, pathways in cancer, MAPK signalling pathway, melanoma, prostate cancer, acute myeloid leukaemia, and colorectal cancer, among others. Previous studies have confirmed the reliability of our analysis results. For example, H2AFY has a significant correlation with lipid metabolism-related HCC [40], as it can also alter the lipid metabolism of HCC cells and allow tumour cells to be more adaptable to the changing microenvironment [23]. Some evidence indicates that H2AFY is a new fusion gene companion for MECOM gene in patients with AML and may promote the development of AML, but the exact mechanism is unclear [28]. H2AFY has also been reported in melanoma, prostate cancer and colorectal cancer [26, 29, 30].

## Conclusions

In general, our study has certain limitations. All analysis results were based on data in the TCGA, and the expression levels of H2AFY protein and mRNA in HCC were not verified in our study. However, our results suggested that H2AFY could be used as a molecular marker for the diagnosis and prognosis of HCC and that a high expression of H2AFY predicted a poor prognosis in patients with HCC.

## Data Availability

The datasets generated during the current study are available in the TCGA database (https:// portal.gdc.cancer.gov/repository).

## Abbreviations

HCC: Hepatocellular carcinoma
TCGA: The cancer genome atlas
GSEA: Gene set enrichment analysis
AFP: α-fetoprotein
EMT: Epithelial mesenchymal transition
MDS: Myelodysplastic syndrome
AML: Acute myeloid leukaemia
NES: Normalized enrichment score
